# Risk factors for subsequent recurrent, relapsing, and repeat peritonitis in peritoneal dialysis

**DOI:** 10.1093/ckj/sfag352

**Published:** 2026-10-05

**Authors:** Hong-Mou Shih, Cheng-Jui Lin, Yi-Zhen Lu, Yu-Wen Hsueh, Chih-Jen Wu, Chi-Feng Pan

**Affiliations:** Division of Nephrology, Department of Internal Medicine, MacKay Memorial Hospital, Taipei, Taiwan; School of Medicine, College of Medicine, MacKay Medical University, Taipei, Taiwan; Graduate Institute of Physiology, College of Medicine, National Taiwan University, Taipei, Taiwan; Division of Nephrology, Department of Internal Medicine, MacKay Memorial Hospital, Taipei, Taiwan; School of Medicine, College of Medicine, MacKay Medical University, Taipei, Taiwan; MacKay Junior College of Medicine, Nursing and Management, Taipei, Taiwan; Division of Nephrology, Department of Internal Medicine, MacKay Memorial Hospital, Taipei, Taiwan; Division of Nephrology, Department of Internal Medicine, MacKay Memorial Hospital, Hsinchu, Taiwan; Division of Nephrology, Department of Internal Medicine, MacKay Memorial Hospital, Taipei, Taiwan; School of Medicine, College of Medicine, MacKay Medical University, Taipei, Taiwan; Department of Medical Research, China Medical University Hospital, China Medical University, Taichung, Taiwan; Division of Nephrology, Department of Internal Medicine, MacKay Memorial Hospital, Taipei, Taiwan; School of Medicine, College of Medicine, MacKay Medical University, Taipei, Taiwan

**Keywords:** manual exchange burden, peritoneal dialysis, recurrent peritonitis, relapsing peritonitis, repeat peritonitis

## Abstract

**Background:**

Peritoneal dialysis (PD)-associated peritonitis may be followed by recurrent, relapsing, or repeat episodes, but factors associated with these subsequent phenotypes remain incompletely defined.

**Methods:**

We conducted a 16-year episode-based study of PD-associated peritonitis. Episodes after which PD was not continued were excluded, and outcomes were defined by the next peritonitis episode. The broader outcome was subsequent recurrent/relapsing/repeat peritonitis, and the narrower outcome was subsequent relapsing/repeat peritonitis. Patient-level and PD prescription-related factors were evaluated for the broader outcome, whereas acute microbiology, antibiotic-management, and severity factors were evaluated for the narrower outcome using logistic generalized estimating equation models. Cross-outcome analyses evaluated both predictor domains against both outcomes.

**Results:**

Among 385 PD-associated peritonitis episodes from 216 patients, 275 episodes from 152 patients were included. Subsequent recurrent/relapsing/repeat peritonitis occurred after 63 episodes. Male sex was associated with lower odds of this outcome, whereas diabetes mellitus, current recurrent/relapsing/repeat phenotype, fewer automated PD treatment days, and greater manual exchange burden were associated with higher odds. However, the two prescription measures were strongly inversely correlated, and neither remained significant when mutually adjusted. Subsequent relapsing/repeat peritonitis occurred after 57 episodes. Ineffective initial antibiotic therapy and delayed adequate antibiotic therapy were associated with higher odds. Organism group and acute severity markers were not significantly associated with this outcome after adjustment. Cross-outcome analyses were broadly compatible with the main findings.

**Conclusions:**

Patient vulnerability, recent episode history, PD prescription-related patterns, and antibiotic-management factors were associated with subsequent peritonitis phenotypes among patients who continued PD. These findings may inform post-peritonitis risk assessment.

## Graphical Abstract

**Graphical Abstract:**
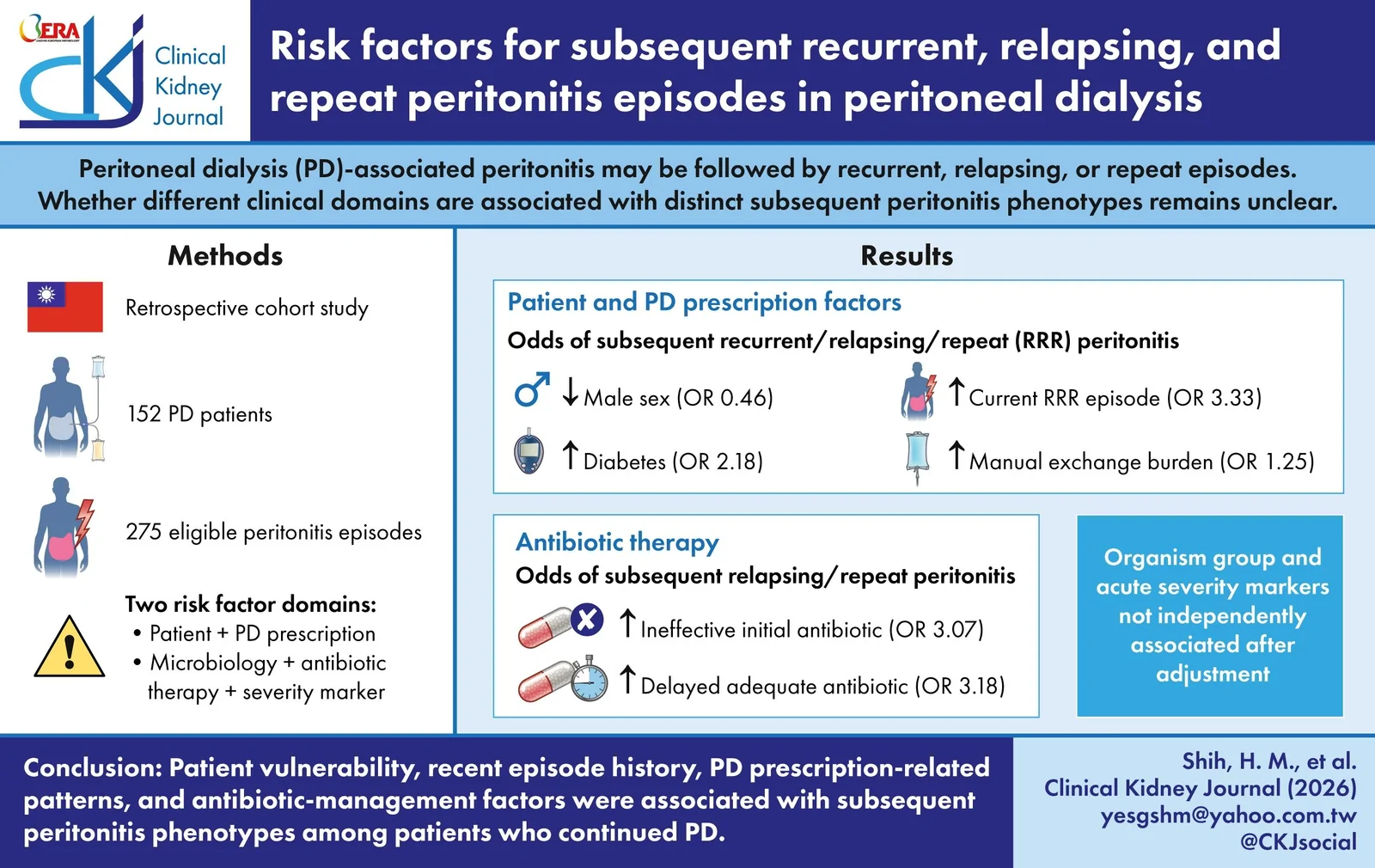

KEY LEARNING POINTS **What was known:**

- Peritoneal dialysis (PD)-associated peritonitis may be followed by recurrent, relapsing, or repeat episodes, with implications for post-peritonitis surveillance, catheter management, and technique survival.
- Previous studies have mainly examined overall peritonitis risk rather than the phenotype of the next episode after each peritonitis episode.
- How patient vulnerability, PD prescription, and acute microbiology or antibiotic-management factors relate to subsequent peritonitis phenotypes remains unclear.

**This study adds:**

- Among episodes after which PD was continued, diabetes mellitus was associated with higher odds and male sex with lower odds of subsequent recurrent/relapsing/repeat peritonitis; current recurrent/relapsing/repeat status was also associated, but should be interpreted primarily as an episode-history marker of ongoing recurrence clustering rather than an independent prognostic factor.
- Fewer automated PD treatment days and greater manual exchange burden were associated with higher odds in separate models, but their independent associations could not be distinguished because the measures were strongly inversely correlated.
- Ineffective initial therapy and delayed adequate therapy were associated with subsequent relapsing/repeat peritonitis, whereas organism group and baseline inflammatory and acute severity markers were not significantly associated after adjustment.

**Potential impact:**

- Post-peritonitis follow-up may benefit from integrating patient vulnerability, PD prescription characteristics, and antimicrobial adequacy rather than relying on organism group or acute severity alone.
- Episodes with ineffective initial therapy or delayed adequate therapy may warrant careful microbiological review, confirmation of treatment response, and closer follow-up.
- Current recurrent/relapsing/repeat status may prompt evaluation for persistent organism-, catheter-, or technique-related sources, while PD prescription complexity may merit individualized assessment.

## INTRODUCTION

Peritoneal dialysis (PD)-associated peritonitis remains one of the most important infectious complications of PD, contributing to hospitalization, catheter loss, transfer to hemodialysis, and mortality [1, 2]. The 2022 International Society for Peritoneal Dialysis (ISPD) recommendations provide standardized definitions for relapsing, recurrent, and repeat peritonitis and advise considering timely catheter removal for these phenotypes [3]. Identifying episodes at risk of clinically related subsequent peritonitis may therefore inform post-peritonitis surveillance, antimicrobial review, and catheter-related decision-making. However, although these phenotypes are often grouped together clinically, they may differ in their underlying mechanisms and predictors [3–5]. Potential predictors can be organized into two clinically guided domains. Patient-level and PD prescription-related factors may reflect host vulnerability, treatment complexity, exchange exposure, or cumulative contamination risk [2, 6], whereas acute episode-level factors may reflect microbiology, treatment adequacy, and inflammatory severity [5].

Previous studies have primarily examined overall peritonitis risk or recurrence, whereas relatively few have evaluated the phenotype of the next episode after each treated peritonitis episode [2, 5, 7–9]. In this episode-based retrospective cohort study, we evaluated patient-level, PD prescription-related, and acute episode-level factors in relation to subsequent peritonitis phenotypes. Primary analyses used clinically guided predictor–outcome pairings, complemented by cross-outcome analyses to assess whether the observed associations extended across outcome definitions.

## MATERIALS AND METHODS

### Study design and population

This was a 16-year episode-based retrospective cohort study of patients receiving PD who developed PD-associated peritonitis at MacKay Memorial Hospital from January 2010 through December 2025. Episodes were retrospectively identified from clinical records, microbiology reports, antibiotic prescription records, and PD prescription data. The unit of analysis was the peritonitis episode because patients could contribute multiple episodes.

PD-associated peritonitis was diagnosed when at least two of the following were present: compatible clinical symptoms or cloudy dialysate, elevated dialysate white blood cell count (WBC) with neutrophil predominance, and positive dialysate culture [3]. At presentation, peritoneal effluent was sampled before antibiotic administration whenever feasible and sent for cell count with differential and microbiological culture. Empirical intraperitoneal antibiotic therapy was initiated after sampling and generally consisted of Gram-positive and Gram-negative coverage with cefazolin plus ceftazidime. Antibiotic therapy was subsequently modified according to culture results, antimicrobial susceptibility testing, and clinical response. When the initially prescribed regimen did not provide adequate organism-level coverage, escalation or modification to an adequate regimen was recorded, and delayed adequate antibiotic therapy was defined as initiation of adequate antimicrobial coverage after the day of presentation. The diagnostic and empirical-treatment approach remained broadly consistent during the study period.

To assess subsequent peritonitis outcomes, the analytic cohort was restricted to episodes after which the patient continued PD. Episodes followed by peritonitis-associated hemodialysis transfer, permanent PD discontinuation, or PD catheter removal without resumption of PD were excluded because patients were no longer at risk for subsequent PD-associated peritonitis.

The study was conducted in accordance with the Declaration of Helsinki and approved by the Institutional Review Board of MacKay Memorial Hospital, which waived the requirement for informed consent because of the retrospective analysis of de-identified clinical data (IRB number: 25MMHIS607e).

### Definitions of subsequent peritonitis outcomes

For each eligible episode, the next PD-associated peritonitis episode was classified according to the ISPD definitions as recurrent, relapsing, repeat, or another subsequent episode [3]. The broader outcome was subsequent recurrent/relapsing/repeat (RRR) peritonitis, defined as the next episode being recurrent, relapsing, or repeat. The narrower outcome was subsequent relapsing/repeat peritonitis. Episodes without a subsequent target event were treated as non-events. Detailed definitions are provided in Table S1.

Each clinically recognized and treated episode, including relapsing episodes, was retained as a separate analytic episode. This episode-based next-event approach differs from the ISPD convention for peritonitis-rate calculation, in which relapsing episodes are treated as extensions of the original episode, because our objective was to characterize the phenotype of the immediately subsequent episode.

### Predictor domains and covariates

Candidate predictors were selected based on clinical relevance and prior literature and grouped into two complementary domains. Within the patient-level and PD prescription-related domain, patient-level factors included age, sex, diabetes mellitus (DM), PD vintage, baseline serum albumin, peritoneal Kt/V, prior exit-site infection, prior tunnel infection, and current RRR status. Prescription-related factors included automated peritoneal dialysis (APD) treatment days, manual exchange burden, glucose load, any 4.25% dextrose use, and icodextrin 7.5% use. These variables were derived from the PD prescription during the 30 days preceding each episode. Manual exchange burden was defined as the cumulative number of prescribed manual exchanges within this window and was modeled per 30 exchanges/month.

The acute episode-level domain included organism group, initial antibiotic adequacy, delayed adequate antibiotic therapy, peripheral blood WBC, initial effluent WBC, C-reactive protein (CRP), and admission status. Organism group was categorized as Gram-positive, Gram-negative, culture-negative, or polymicrobial/fungal/other. Initial antibiotic adequacy was classified as effective, ineffective, or not assessable according to organism-level antimicrobial coverage. Delayed adequate antibiotic therapy was defined as initiation of an adequate antimicrobial regimen after the day of presentation. Admission status was defined as any emergency department visit, hospitalization, or intensive care unit admission related to the episode.

Continuous variables were scaled or transformed for clinical interpretability. Detailed coding, derivation, and scaling are provided in Table S2.

### Statistical analysis

Baseline characteristics of eligible peritonitis episodes were summarized according to the presence or absence of subsequent RRR peritonitis. Continuous variables were presented as medians with interquartile ranges, and categorical variables were presented as numbers and percentages. Available denominators were reported for variables with missing data. To characterize potential selection related to continued PD and catheter management, analytic-cohort disposition and catheter-management pathways in the full source cohort were summarized descriptively according to current RRR status. Characteristics of excluded episodes, including refractory status, were also summarized, with microbiology and disposition described for excluded refractory episodes.

Because patients could contribute more than one peritonitis episode, logistic generalized estimating equation (GEE) models were used to account for patient-level clustering. Each candidate predictor was evaluated in a separate logistic GEE model with a binomial distribution, logit link, independence working correlation structure, and robust standard errors. Results were reported as odds ratios (ORs) with 95% confidence intervals (CIs).

For the broader RRR outcome, patient-level and PD prescription-related factors were evaluated in separate logistic GEE models. Adjusted models included age, sex, DM, and current RRR status, except when the covariate was the predictor of interest. Because APD treatment days and manual exchange burden were expected to represent related aspects of PD prescription, their correlation was assessed using Spearman’s correlation. We additionally performed a mutually adjusted GEE model including both variables. For the narrower relapsing/repeat outcome, acute episode-level factors were evaluated in separate logistic GEE models. Adjusted models included peripheral blood WBC, log₂-transformed initial effluent WBC, and admission status, except when the covariate was the predictor of interest. Initial antibiotic adequacy and delayed adequate antibiotic therapy were analyzed in separate models.

To assess whether the original predictor–outcome pairing influenced the findings, we performed additional cross-outcome analyses. All patient-level and PD prescription-related predictors were evaluated against both the broader subsequent RRR and narrower subsequent relapsing/repeat outcomes, and all acute episode-level predictors were similarly evaluated against both outcomes. Within each predictor domain, the same prespecified adjustment set was applied to the two outcome definitions.

Additional sensitivity analyses included organism-restricted and endpoint-restricted analyses of acute episode-level predictors and calendar-period-adjusted models for both predictor domains. Calendar period was included to account for potential temporal changes in patient and episode characteristics, culture yield, and empirical treatment practices.

Missing data were handled using model-specific complete-case analysis, with missingness summarized in Table S3. Peripheral blood WBC and CRP availability was related to acute-care presentation; therefore, a reduced-adjustment sensitivity analysis was performed to assess the influence of missing peripheral blood WBC. Culture-negative episodes were excluded from antibiotic-specific analyses because organism-level adequacy and time to adequate therapy could not be determined; these values were not imputed.

Because follow-up duration varied across episodes, we performed complementary time-at-risk analyses. For each outcome, follow-up ended at the first occurrence of the target peritonitis event, a competing event, or censoring. Competing events included death, kidney transplantation, permanent transfer to hemodialysis or other permanent discontinuation of PD, and a subsequent peritonitis episode not meeting the target outcome definition. Transfer to another hospital was treated as a censoring event, and follow-up was administratively censored on 31 December 2025. Incidence rates were expressed per 100 episode-years with exact Poisson 95% CIs.

Because the study was exploratory and hypothesis-generating, no formal adjustment for multiple comparisons was applied. All tests were two-sided, and *P* < .05 was considered statistically significant. Effect estimates, 95% CIs, *P* values, and consistency across sensitivity analyses were interpreted collectively. Analyses were performed using IBM SPSS Statistics version 23 (IBM Corp., Armonk, NY, USA).

## RESULTS

During the 16-year study period from 2010 to 2025, the PD unit cared for ∼530 patients receiving PD, including 376 incident PD patients, and contributed 2131.8 patient-years of PD follow-up. A total of 385 PD-associated peritonitis episodes from 216 patients met the diagnostic criteria, corresponding to an overall peritonitis rate of 0.172 episodes per patient-year. Culture-negative peritonitis accounted for 68/385 episodes (17.7%). Across calendar periods, the peritonitis rate was 0.179, 0.156, and 0.180 episodes per patient-year in 2010–2015, 2016–2020, and 2021–2025, respectively, and the corresponding culture-negative proportions were 15.4%, 15.8%, and 23.5%. These unit-level metrics for the full source cohort are summarized in Table S4. After excluding 110 episodes after which patients were no longer receiving PD, 275 episodes from 152 patients were included in the subsequent peritonitis analyses (Fig. 1).

**Figure 1:**
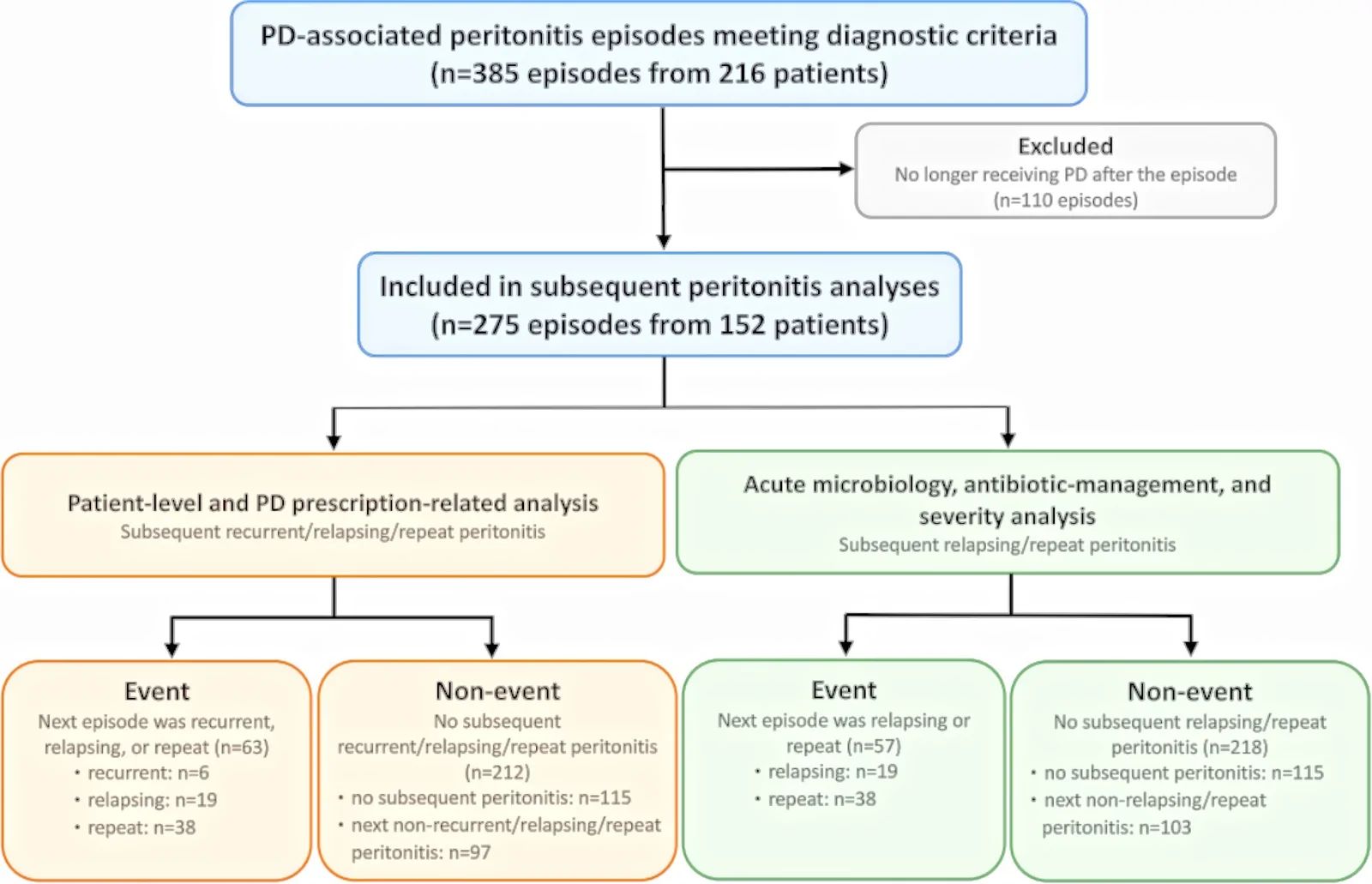
Study flow and definition of subsequent peritonitis outcomes. Among 385 PD-associated peritonitis episodes meeting diagnostic criteria, 110 episodes were excluded because patients were no longer receiving PD after the episode. The remaining 275 episodes were included in the subsequent peritonitis analyses. Outcomes were defined by the next PD-associated peritonitis episode. The broader outcome was subsequent RRR peritonitis, and the narrower outcome was subsequent relapsing/repeat peritonitis.

Among the 65 episodes with a current RRR phenotype in the full source cohort, 6 were recurrent, 19 were relapsing, and 40 were repeat episodes. Of these, 14 were excluded because PD was not subsequently continued, whereas 51 were retained in the analytic cohort. Among current RRR episodes, peritonitis-associated catheter removal was documented in 13 of 14 excluded episodes (92.9%) and in none of the 51 retained episodes. Two catheter-removal episodes without a current RRR phenotype were retained after catheter reinsertion and resumption of PD. Overall, 105/110 (95.5%) excluded episodes underwent peritonitis-associated catheter removal. Among the 44 excluded episodes with assessable refractory status, 42 (95.5%) were refractory. Among these 42 refractory episodes, Gram-negative organisms were most common (22/42 [52.4%]); 41 (97.6%) underwent peritonitis-associated catheter removal and 5 (11.9%) had peritonitis-associated death. The detailed subtype distribution, catheter-management pathways, and characteristics of excluded episodes, including the refractory subgroup, are summarized in Table S5.

Among the 275 eligible episodes, the median follow-up duration was 294 days (interquartile range [IQR], 66.5–826), corresponding to 434.5 episode-years at risk. Subsequent RRR peritonitis occurred after 63 episodes, and subsequent relapsing/repeat peritonitis occurred after 57 episodes, corresponding to incidence rates of 14.5 (95% CI, 11.1–18.6) and 13.1 (95% CI, 9.9–17.0) events per 100 episode-years, respectively. During follow-up, 32 episodes were followed by death, 8 by kidney transplantation, and 37 by permanent hemodialysis transfer or PD discontinuation before a subsequent peritonitis episode. Complete follow-up disposition and organism-specific time-based incidence rates are presented in Table S6.

Baseline characteristics according to subsequent RRR peritonitis are summarized in Table 1. Among patient-level and PD prescription-related factors, adjusted models showed that male sex was associated with lower odds of subsequent RRR peritonitis, whereas DM and a current recurrent/relapsing/repeat phenotype were associated with higher odds (Table 2 and Fig. 2A). Fewer APD treatment days and greater manual exchange burden were also associated with higher odds in separate models. However, these prescription measures were strongly inversely correlated (Spearman’s *ρ* = −0.882; *P* < .001), and neither remained significant when mutually adjusted (Table S7). Peritoneal Kt/V showed a non-significant positive association.

**Figure 2:**
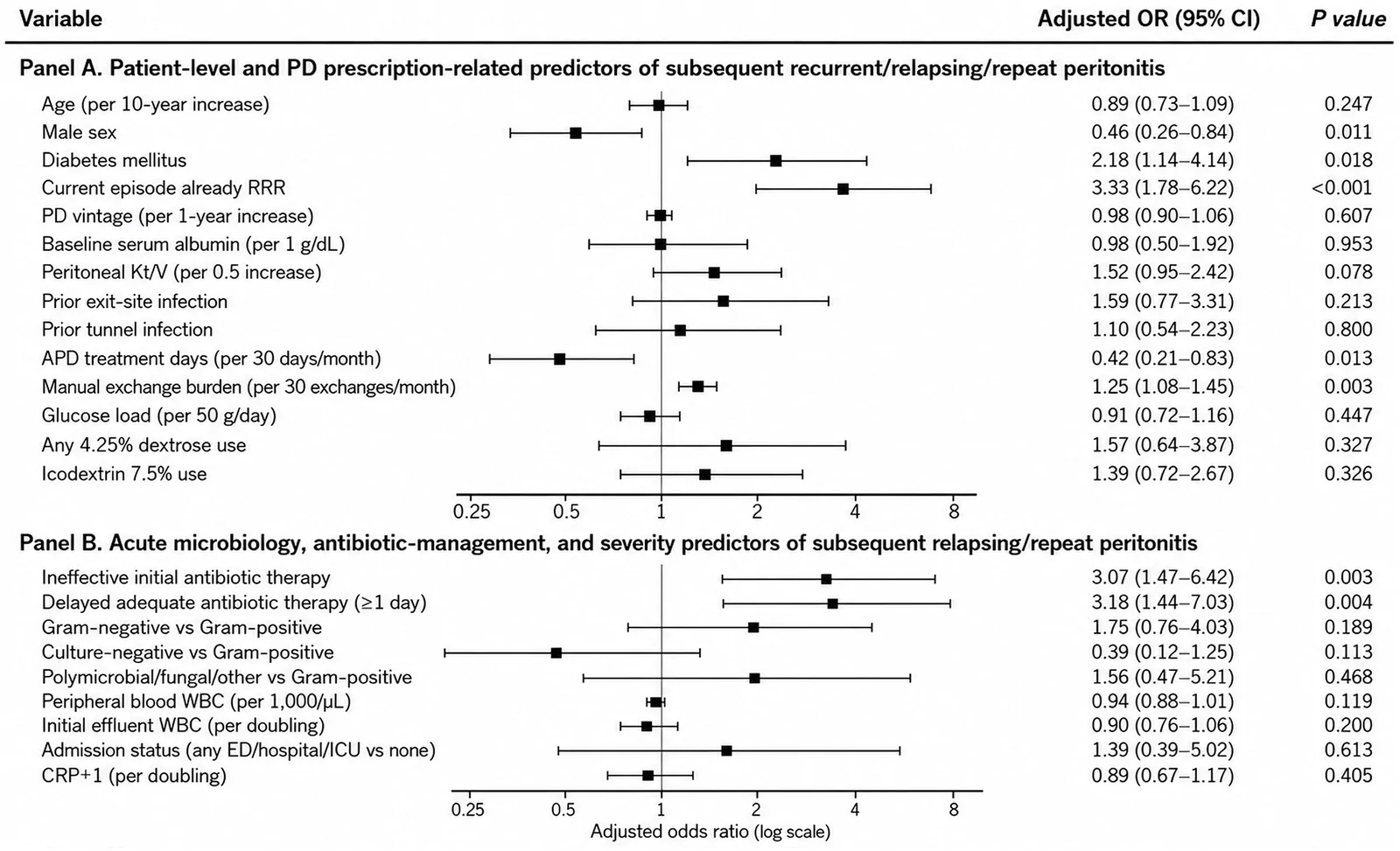
Forest plot of adjusted associations with subsequent peritonitis outcomes. Panel (A) shows adjusted associations of patient-level and PD prescription-related factors with subsequent RRR peritonitis. APD treatment days and manual exchange burden in panel (A) were evaluated in separate models because of their strong inverse correlation; mutually adjusted estimates are presented in Table S7. Panel (B) shows adjusted associations of acute microbiology, antibiotic-management, and severity factors with subsequent relapsing/repeat peritonitis. ORs were estimated using logistic GEE models and are displayed on a logarithmic scale with 95% CIs. The vertical reference line indicates an OR of 1.0.

**Table 1:** Characteristics of eligible peritonitis episodes according to the presence of subsequent RRR peritonitis (*N* = 275).

Characteristic	Available *N*	No subsequent recurrent/relapsing/repeat (*N* = 212)	Subsequent recurrent/relapsing/repeat (*N* = 63)
Patient-level factors			
Age, years	275	55.4 (43.9–61.7)	54.3 (45.8–59.2)
Male sex	275	112/212 (52.8)	22/63 (34.9)
Diabetes mellitus	275	39/212 (18.4)	18/63 (28.6)
PD vintage, years	274	4.10 (1.96–7.13)	5.09 (2.43–7.22)
Baseline serum albumin, g/dL	245	3.9 (3.5–4.2)	3.8 (3.5–4.1)
Peritoneal Kt/V	253	1.83 (1.53–2.12)	2.06 (1.68–2.43)
Prior exit-site infection	275	33/212 (15.6)	15/63 (23.8)
Prior tunnel infection	275	10/212 (4.7)	4/63 (6.3)
PD modality and prescription factors			
APD treatment days, days/month	275	30.0 (0.0–30.0)	20.0 (0.0–30.0)
Manual exchange burden, exchanges/month	275	12.0 (0.0–120.0)	60.0 (26.0–120.0)
Glucose load, g/day	275	187.5 (137.5–250.0)	178.8 (150.0–230.5)
Any 4.25% dextrose use	275	21/212 (9.9)	9/63 (14.3)
Icodextrin 7.5% use	275	125/212 (59.0)	45/63 (71.4)
Microbiology and antibiotic factors			
Organism group			
Gram-positive	275	94/212 (44.3)	30/63 (47.6)
Gram-negative	275	47/212 (22.2)	23/63 (36.5)
Culture-negative	275	53/212 (25.0)	4/63 (6.3)
Polymicrobial/fungal/other	275	18/212 (8.5)	6/63 (9.5)
Initial antibiotic adequacy			
Effective	274	123/211 (58.3)	34/63 (54.0)
Ineffective	274	35/211 (16.6)	25/63 (39.7)
Not assessable	274	53/211 (25.1)	4/63 (6.3)
Delayed adequate antibiotics (≥1 day after presentation)	212	32/155 (20.6)	24/57 (42.1)
Time to adequate antibiotics, days	212	0.0 (0.0–0.0)	0.0 (0.0–3.0)
Acute episode severity markers			
Peripheral blood WBC, cells/µL	192	10 550 (7800–14 050)	9100 (6500–12 500)
Initial effluent WBC, cells/µL	273	1756 (698–3756)	1870 (630–3780)
CRP, mg/dL	153	5.02 (1.13–12.48)	4.31 (1.15–15.77)
Any ED/hospital/ICU	275	141/212 (66.5)	43/63 (68.3)

Note: Values are presented as median (interquartile range) or *n*/*N* (%). Subsequent recurrent/relapsing/repeat peritonitis was defined according to the classification of the next PD-associated peritonitis episode. Episodes with no subsequent peritonitis or with a subsequent non-RRR episode were included in the no subsequent recurrent/relapsing/repeat group.

Abbreviations: APD, automated peritoneal dialysis; CRP, C-reactive protein; ED, emergency department; ICU, intensive care unit; PD, peritoneal dialysis; RRR, recurrent/relapsing/repeat peritonitis; WBC, white blood cell count.

**Table 2:** Patient-level and PD prescription-related predictors of subsequent RRR peritonitis.

Variable	Crude OR (95% CI)	Crude *P*	Adjusted OR (95% CI)	Adjusted *P*
Age (per 10-year increase)	0.98 (0.81–1.17)	.798	0.89 (0.73–1.09)	.247
Male sex (vs female)	0.48 (0.25–0.94)	.031	0.46 (0.26–0.84)	.011
Diabetes mellitus	1.77 (0.85–3.70)	.126	2.18 (1.14–4.14)	.018
Current episode already RRR	3.78 (2.05–6.95)	<.001	3.33 (1.78–6.22)	<.001
PD vintage (per 1-year increase)	1.02 (0.94–1.11)	.648	0.98 (0.90–1.06)	.607
Baseline serum albumin (per 1 g/dL increase)	0.80 (0.44–1.48)	.480	0.98 (0.50–1.92)	.953
Peritoneal Kt/V (per 0.5 increase)	1.84 (1.25–2.69)	.002	1.52 (0.95–2.42)	.078
Prior exit-site infection	1.70 (0.71–4.06)	.236	1.59 (0.77–3.31)	.213
Prior tunnel infection	1.37 (0.55–3.41)	.499	1.10 (0.54–2.23)	.800
APD treatment days (per 30 days/month)	0.43 (0.21–0.87)	.020	0.42 (0.21–0.83)	.013
Manual exchange burden (per 30 exchanges/month)	1.26 (1.09–1.47)	.003	1.25 (1.08–1.45)	.003
Glucose load (per 50 g/day)	0.90 (0.73–1.13)	.369	0.91 (0.72–1.16)	.447
Any 4.25% dextrose use	1.52 (0.68–3.37)	.308	1.57 (0.64–3.87)	.327
Icodextrin 7.5% use	1.74 (0.86–3.51)	.122	1.39 (0.72–2.67)	.326

Note: The broader outcome was defined as the next peritonitis episode being classified as recurrent, relapsing, or repeat. Episodes with no subsequent peritonitis or a subsequent non-RRR episode were treated as non-events. Each predictor was evaluated in a separate logistic GEE model with binomial distribution, logit link, independence working correlation structure, patient-level clustering, and robust standard errors. Adjusted models included age, sex, diabetes mellitus, and current RRR status, except that the predictor itself was omitted from the adjustment set when applicable.

Abbreviations: APD, automated peritoneal dialysis; CI, confidence interval; GEE, generalized estimating equations; OR, odds ratio; PD, peritoneal dialysis; RRR, recurrent/relapsing/repeat peritonitis.

Among acute microbiology, antibiotic-management, and severity factors, ineffective initial antibiotic therapy was associated with higher odds of subsequent relapsing/repeat peritonitis compared with effective initial therapy (adjusted OR, 3.07; 95% CI, 1.47–6.42; *P* = .003). Delayed adequate antibiotic therapy was also associated with higher odds compared with same-day adequate therapy (adjusted OR, 3.18; 95% CI, 1.44–7.03; *P* = .004). Organism group and acute severity markers were not significantly associated with subsequent relapsing/repeat peritonitis after adjustment (Table 3 and Fig. 2B).

**Table 3:** Acute microbiology, antibiotic-management, and severity predictors of subsequent relapsing/repeat peritonitis.

Variable	Crude OR (95% CI)	Crude *P*	Adjusted OR (95% CI)	Adjusted *P*
Organism group				
Gram-positive	Reference		Reference	
Gram-negative vs Gram-positive	1.62 (0.76–3.42)	.210	1.75 (0.76–4.03)	.189
Culture-negative vs Gram-positive	0.28 (0.10–0.80)	.017	0.39 (0.12–1.25)	.113
Polymicrobial/fungal/other vs Gram-positive	1.26 (0.41–3.85)	.690	1.56 (0.47–5.21)	.468
Initial antibiotic adequacy				
Effective	Reference		Reference	
Ineffective vs effective	2.94 (1.55–5.60)	.001	3.07 (1.47–6.42)	.003
Time to adequate antibiotics				
Same-day adequate antibiotics	Reference		Reference	
Delayed ≥1 day after presentation vs same-day	3.19 (1.62–6.27)	<.001	3.18 (1.44–7.03)	.004
Acute severity markers				
Peripheral blood WBC (per 1000/μL increase)	0.95 (0.88–1.02)	.163	0.94 (0.88–1.01)	.119
Initial effluent WBC (per doubling)	0.94 (0.80–1.11)	.468	0.90 (0.76–1.06)	.200
Admission status (any ED/hospital/ICU vs none)	0.99 (0.53–1.83)	.965	1.39 (0.39–5.02)	.613
CRP + 1 (per doubling)	0.88 (0.67–1.16)	.370	0.89 (0.67–1.17)	.405

Note: The narrower outcome was defined as the next peritonitis episode being classified as relapsing or repeat. Episodes with no subsequent peritonitis or a subsequent non-relapsing/repeat episode were treated as non-events. All models were fitted using GEE with a binomial distribution, logit link, independence working correlation structure, and robust standard errors. Each predictor was evaluated in a separate GEE logistic regression model. Adjusted models included peripheral blood WBC, log_2_-transformed initial effluent WBC, and admission status, except that the covariate was omitted from the adjustment set when it was the predictor of interest.

Abbreviations: CI, confidence interval; CRP, C-reactive protein; ED, emergency department; GEE, generalized estimating equations; ICU, intensive care unit; OR, odds ratio; WBC, white blood cell count.

Cross-outcome analyses showed substantial overlap between predictor domains. Patient-level and PD prescription-related findings were generally similar for the narrower relapsing/repeat outcome, while ineffective initial therapy and delayed adequate therapy were also associated with the broader RRR outcome. Acute severity markers were not significantly associated with either outcome (Table S8).

Additional sensitivity analyses of acute episode-level factors are summarized in Table S9. When analyses were restricted to Gram-positive or Gram-negative episodes combined, ineffective initial antibiotic therapy and delayed adequate antibiotic therapy remained associated with subsequent relapsing/repeat peritonitis. Organism-specific subgroup estimates were imprecise, particularly for Gram-negative episodes. In endpoint-restricted analyses, these associations were more apparent for repeat-only than for relapsing-only peritonitis. In sensitivity analyses omitting peripheral blood WBC from the adjustment set, ineffective initial therapy and delayed adequate therapy remained associated with subsequent relapsing/repeat peritonitis, whereas initial effluent WBC, CRP, and admission status remained non-significant (Table S10).

To account for potential temporal changes in patient and episode characteristics, culture yield, and empirical treatment practices, we additionally adjusted the main models for calendar period, categorized as 2010–2015, 2016–2020, and 2021–2025. The main associations were generally directionally consistent after calendar-period adjustment. Male sex, DM, current RRR phenotype, manual exchange burden, ineffective initial antibiotic therapy, and delayed adequate antibiotic therapy remained associated with their respective outcomes (Table S11).

## DISCUSSION

In this 16-year episode-based retrospective cohort study of PD-associated peritonitis, we evaluated clinical factors associated with subsequent peritonitis phenotypes among patients who continued PD. Patient vulnerability, recent episode history, and PD prescription patterns were associated with subsequent RRR peritonitis, while ineffective initial antibiotic therapy and delayed adequate therapy were associated with subsequent relapsing/repeat peritonitis. These findings suggest that post-peritonitis risk assessment may benefit from considering patient-, prescription-, and episode-level factors together.

Among patient-level factors, DM was associated with higher odds of subsequent RRR peritonitis, whereas male sex was associated with lower odds. DM may reflect host vulnerability, comorbidity, and care complexity. Its association with PD-related peritonitis has varied across cohorts and treatment eras and may be influenced by contemporary PD systems, infection-prevention practices, patient training, and assisted-care support [10–14]. Accordingly, DM should be interpreted as a risk marker rather than an isolated causal factor. Male sex was associated with lower odds of both subsequent peritonitis outcomes. This finding was unexpected, and sex-related associations with PD peritonitis have not been consistent across previous studies. The apparent protective association therefore warrants cautious interpretation and may reflect population-specific factors or residual confounding rather than a biological protective effect of male sex [4].

The association between a current RRR phenotype and subsequent RRR peritonitis should be interpreted cautiously. Because current RRR status is defined in relation to the preceding episode, whereas subsequent RRR status is defined in relation to the following episode, these adjacent classifications are inherently serially dependent, particularly within clusters of relapsing or repeat episodes. The observed association may therefore reflect ongoing clustering of related episodes, together with persistent patient-, organism-, catheter-, or technique-related susceptibility, rather than independent prognostic information provided by the current RRR phenotype itself. Although GEE accounted for repeated episodes within patients, it cannot eliminate this temporal and definitional dependence. Catheter management may further influence whether patients remain at risk for another PD-associated peritonitis episode. Notably, all 13 current RRR episodes with peritonitis-associated catheter removal were excluded because PD was not subsequently continued, whereas none of the 51 current RRR episodes retained in the analytic cohort underwent peritonitis-associated catheter removal. Consequently, the observed association between current and subsequent RRR peritonitis pertains specifically to patients who remained on PD after the current episode. Although catheter-removal status was identifiable, the clinical rationale for catheter removal or retention and more detailed catheter-management pathways were not consistently available. Current RRR status should therefore be regarded primarily as an episode-history marker of recurrence clustering rather than an independent effect of the RRR phenotype or catheter-management strategy.

PD prescription-related findings appeared to reflect a shared modality- or prescription-related pattern rather than clearly separable effects of APD treatment days and manual exchange burden. Although fewer APD treatment days and greater manual exchange burden were associated with higher odds of subsequent RRR peritonitis in separate adjusted models, their independent associations could not be distinguished when both variables were included simultaneously because of strong collinearity and unstable estimates. These findings may reflect differences in exchange frequency, cumulative connection exposure, treatment complexity, opportunities for contamination, or underlying patient characteristics rather than direct effects of the prescribed modality or exchange frequency. This cautious interpretation is consistent with previous studies comparing APD and continuous ambulatory peritoneal dialysis (CAPD), which have reported mixed findings regarding peritonitis risk [6, 15, 16]. Such heterogeneity may reflect differences in connection systems, patient training, local practice patterns, patient populations, and changes in PD technology over time [2, 17, 18]. Our study further differed from studies of incident PD patients or first peritonitis episodes because all eligible episodes occurred in patients who had already developed PD-associated peritonitis and subsequently remained on PD. This selected population may have been enriched for unmeasured technique-related, environmental, or treatment-related vulnerabilities, potentially increasing the clinical relevance of prescription-related measures in our episode-based analysis. Manual exchange burden may therefore function primarily as a marker of cumulative connection exposure and treatment complexity, rather than as an isolated causal factor. Another finding in this domain that warrants cautious interpretation is the positive peritoneal Kt/V estimate. This estimate should not be taken to indicate harm from greater solute clearance; rather, it may reflect peritoneal transport characteristics, prescription intensity, body size, residual confounding, or chance and should be considered hypothesis-generating.

Among acute episode-level factors, ineffective initial therapy and delayed adequate therapy were associated with subsequent relapsing/repeat peritonitis, possibly reflecting antimicrobial resistance, biofilm, catheter-related persistence, or incomplete microbial eradication [3, 19, 20]. However, antibiotic adequacy was determined retrospectively and could not be assessed in culture-negative episodes. These variables may therefore reflect organism characteristics and difficulty of eradication as well as treatment decisions and should be regarded as episode-level risk markers rather than causal treatment effects. Interestingly, the antibiotic-management associations were more apparent for repeat-only than for relapsing-only peritonitis. Persistent catheter biofilm or other microbial reservoirs could contribute to later same-organism re-emergence, but the relapsing-only analyses included fewer events and were imprecise. The similar point estimates for ineffective initial therapy across the two endpoints further argue against a clear difference between relapsing and repeat peritonitis. This pattern suggests that associations with antibiotic-management factors may not be confined to the immediate 4-week relapse window, although the endpoint-specific analyses were imprecise and exploratory.

Organism-specific findings also require cautious interpretation. The crude incidence of subsequent RRR peritonitis was higher after Gram-negative than after Gram-positive episodes (26.8 vs 14.4 events per 100 episode-years), which was directionally consistent with the adjusted logistic estimate (adjusted OR, 1.77; 95% CI, 0.80–3.90). The incidence analysis incorporated episode-time at risk and competing or censoring events, whereas the logistic GEE model assessed the next-episode phenotype without explicitly modeling event time. Thus, the findings suggest a higher crude time-based incidence after Gram-negative episodes, although the adjusted estimate was insufficiently precise to establish an independent association. This direction is consistent with previous studies showing poorer outcomes after Gram-negative peritonitis; however, those studies primarily evaluated episode resolution, hospitalization, catheter loss or removal, technique failure, transfer to hemodialysis, or mortality rather than the phenotype of a subsequent peritonitis episode [21, 22].

Culture-negative episodes also require additional caution because the outcome definition itself restricts their classification. Repeat peritonitis requires recurrence with the same identified organism, whereas relapsing peritonitis allows one of the paired episodes to be culture-negative. Consequently, the low time-based incidence after culture-negative episodes (3.8 events per 100 episode-years) and the lower adjusted odds for the broader RRR outcome (adjusted OR, 0.29; 95% CI, 0.09–0.93) may partly reflect this definitional feature rather than a biologically protective association. The corresponding estimate for the narrower relapsing/repeat outcome was also below unity but was imprecise (adjusted OR, 0.39; 95% CI, 0.12–1.25).

With respect to temporal trends, the higher culture-negative proportion observed in 2021–2025 (23.5%) may reflect unmeasured changes in pre-culture antibiotic exposure, specimen collection or transport, microbiological processing, or the timing of presentation relative to symptom onset, despite a broadly consistent diagnostic and empirical-treatment approach during the study period. These factors were not systematically captured, and the cause of the temporal increase could not be determined [3].

Inflammatory and acute severity markers were not significantly associated with subsequent relapsing/repeat peritonitis. However, these findings do not establish that baseline inflammatory intensity is uninformative, particularly because peripheral blood WBC and CRP were selectively measured during acute-care evaluation and missingness may have reduced statistical power. Prior studies have examined treatment-response measures, including effluent WBC kinetics, and emerging effluent biomarkers such as interleukin-6, matrix metalloproteinase-8, and bacteria-derived DNA as potential complementary markers of local inflammatory activity or treatment response [23–27]. These measures were not routinely available in our cohort, and their prognostic value relative to conventional baseline markers requires prospective evaluation.

Cross-outcome analyses showed substantial overlap, indicating that the two-domain framework was clinically guided rather than evidence of distinct outcome-specific mechanisms; sensitivity analyses were broadly compatible with the main findings, although subgroup and endpoint-specific estimates were imprecise.

Among patients who continued PD after peritonitis, our findings may help guide risk-informed post-peritonitis surveillance and management. Episodes with ineffective initial antibiotic therapy or delayed adequate therapy may warrant careful review of culture and susceptibility results, confirmation of clinical and dialysate response, and closer follow-up. However, the observational design does not establish that modifying empirical therapy would prevent subsequent events. DM may identify patients at higher risk, whereas a current RRR phenotype may mainly indicate that the patient may already be within a cluster of consecutive related episodes. The latter may warrant review for persistent organism-, catheter-, or technique-related sources, although its association with subsequent RRR peritonitis is partly attributable to serial dependence between consecutive episode classifications. Catheter management should remain guided by the clinical presentation and ISPD recommendations, including catheter removal when indicated. For patients who continue PD, structured technique review, reinforcement of exchange procedures, and closer monitoring may help address potentially modifiable risks. In patients with higher manual exchange burden, reviewing the necessity and frequency of manual exchanges or simplifying the PD prescription when clinically feasible may also be reasonable. Recurrent or severe peritonitis may also contribute to cumulative peritoneal membrane injury and has been associated with encapsulating peritoneal sclerosis, further underscoring the importance of preventing additional episodes and monitoring long-term peritoneal membrane health.

Several limitations should be acknowledged. First, this was a retrospective single-center study, and residual confounding and limited generalizability cannot be excluded. Because the analysis was restricted to episodes after which PD was continued, most refractory or technique-failure episodes were excluded, selecting a cohort with more favorable immediate outcomes. Accordingly, the findings apply primarily to patients who remained on PD after peritonitis. Although the time-at-risk analyses summarized incidence rates over follow-up, the primary logistic GEE models assessed the phenotype of the next peritonitis episode and did not fit time-to-event regression models or estimate competing-risk cumulative incidence.

Second, selective availability of peripheral blood WBC and CRP, together with incomplete assessability of initial antibiotic adequacy and time to adequate therapy, may have reduced precision. Antibiotic-specific analyses were necessarily limited to episodes in which organism-level antimicrobial coverage could be assessed, potentially introducing selection related to culture yield. Moreover, because ineffective initial therapy and delayed adequate therapy were defined retrospectively using microbiological and susceptibility information, they may have reflected underlying organism resistance, virulence, biofilm propensity, difficulty of eradication, and the timing of microbiological results, in addition to treatment decisions. Residual confounding by organism characteristics and treatment-context factors therefore cannot be excluded, and these associations should not be interpreted causally.

Third, detailed information on exchange technique, retraining, home environment, caregiver involvement, catheter biofilm, adherence to antibiotic protocols, and catheter removal or reinsertion was not consistently available. Concurrent exit-site infection at the time of the episode under analysis was also not consistently captured and could not be evaluated separately from a documented history of prior exit-site infection. In addition, PD prescription variables may have reflected underlying patient characteristics, clinical status, and treatment selection rather than independent causal effects.

Finally, several predictors and outcomes were evaluated without formal adjustment for multiple comparisons. Some nominally significant or borderline findings, particularly those with *P* values close to .05 or wide CIs, may therefore represent chance associations and should be interpreted cautiously.

## CONCLUSIONS

Among patients who remained on PD after peritonitis, patient-level characteristics, recent episode history, and PD prescription-related markers were associated with subsequent RRR peritonitis, while ineffective initial antibiotic therapy and delayed adequate therapy were associated with subsequent relapsing/repeat peritonitis. These findings may inform post-peritonitis risk assessment by integrating patient vulnerability, prior episode patterns, PD prescription characteristics, and antibiotic-management factors, while recognizing that the observed associations do not establish independent causal effects. Given the exploratory design, multiple comparisons, and limited precision of some estimates, these findings should be considered hypothesis-generating and require confirmation in independent cohorts.

## Supplementary Material

sfag352_Supplemental_File

## Data Availability

The individual-level data supporting the findings of this study are not publicly available because they contain potentially identifiable clinical information and are subject to patient privacy regulations and institutional restrictions imposed by the Institutional Review Board of MacKay Memorial Hospital. De-identified data or additional analyses may be made available from the corresponding author upon reasonable request and with appropriate institutional approval.
