# Supplementary material for: Risk factors for subsequent recurrent, relapsing, and repeat peritonitis in peritoneal dialysis
Source: Clin Kidney J. 2026 Oct 5;19(10):sfag352. doi: 10.1093/ckj/sfag352 (PMC13696888; doi:10.1093/ckj/sfag352)
Supplement: Supplementary file 1 [file sfag352_supplemental_file.pdf]

Supplementary Material

**Supplementary Table S1. Outcome definitions and analytic denominator**	
**Term**	**Definition in this study**
Episode under analysis	An eligible PD-associated peritonitis episode used as the starting point for classification of the next PD-associated peritonitis episode.
Analytic denominator	PD-associated peritonitis episodes after which the patient continued PD and remained at risk for a subsequent PD-associated peritonitis episode. Episodes followed by peritonitis-associated hemodialysis transfer, permanent PD discontinuation, or PD catheter removal without resumption of PD were excluded.
Subsequent peritonitis	The next PD-associated peritonitis episode occurring after the episode under analysis. Only the next episode was used to define the subsequent peritonitis phenotype.
Relapsing peritonitis	A subsequent episode occurring within 4 weeks after completion of therapy for the preceding episode, caused by the same organism, or with one of the paired episodes being culture-negative.
Repeat peritonitis	A subsequent episode occurring more than 4 weeks after completion of therapy for the preceding episode and caused by the same organism.
Recurrent peritonitis	A subsequent episode occurring within 4 weeks after completion of therapy for the preceding episode and caused by a different organism.

Note: Definitions are episode-based. For each eligible episode, only the next PD-associated peritonitis episode occurring after the episode under analysis was used to define the subsequent peritonitis phenotype. Episodes followed by peritonitis-associated hemodialysis transfer, permanent PD discontinuation, or PD catheter removal without resumption of PD were excluded because patients were no longer receiving PD and were therefore not at risk for a subsequent PD-associated peritonitis episode.

Abbreviations: PD, peritoneal dialysis.

Supplementary Table S2. Variable definitions	
**Variable**	**Coding / derivation**
Initial antibiotic adequacy	effective / ineffective / not assessable
Delayed adequate antibiotics	adequate antibiotic started ≥1 day after presentation
Effluent WBC	$\log_2$-transformed / per doubling
CRP	$\log_2$(CRP+1) / per doubling
Peritoneal Kt/V	PD-fluid/peritoneal Kt/V modeled per 0.5-unit increase
APD treatment days	Number of APD treatment days in the 30-day prescription window; modeled per 30 days/month
Manual exchange burden	Number of prescribed manual exchanges in the 30-day prescription window; modeled per 30 exchanges/month
Glucose load	per 50 g/day
Any 4.25% dextrose use	any 4.25% total volume >0
Icodextrin use	any 7.5% PD fluid volume >0
Current episode already RRR	episode under analysis classified as recurrent, relapsing, or repeat
Prior exit-site infection	history before episode under analysis
Prior tunnel infection	history before episode under analysis

Note: Variable definitions and transformations reflect how variables were coded for the main and supplementary analyses. Effluent WBC was modeled after $\log_2$ transformation and interpreted per doubling. CRP was modeled as $\log_2$(CRP+1) and interpreted per doubling of CRP+1. Peritoneal Kt/V, APD treatment days, manual exchange burden, and glucose load were scaled as shown in the table for model interpretation.

Abbreviations: APD, automated peritoneal dialysis; CRP, C-reactive protein; Kt/V, urea clearance index; PD, peritoneal dialysis; RRR, recurrent/relapsing/repeat peritonitis; WBC, white blood cell count.

Supplementary Table S3. Missing or not-assessable data				
Variable	Analysis cohort N	Available / assessable n	Missing / not assessable n	Missing / not assessable %
**Patient-level factors**				
Age	275	275	0	0.0
Sex	275	275	0	0.0
Diabetes mellitus	275	275	0	0.0
PD vintage	275	274	1	0.4
Baseline serum albumin	275	245	30	10.9
Peritoneal Kt/V	275	253	22	8.0
**Patient / catheter history**				
Prior exit-site infection	275	275	0	0.0
Prior tunnel infection	275	275	0	0.0
**PD modality and prescription**				
APD treatment days	275	275	0	0.0
Manual exchange burden	275	275	0	0.0
Glucose load	275	275	0	0.0
Any 4.25% dextrose use	275	275	0	0.0
Icodextrin 7.5% use	275	275	0	0.0
**Episode history**				
Current episode already RRR	275	275	0	0.0
**Microbiology and antibiotics**				
Organism group	275	275	0	0.0
Initial antibiotic adequacy	275	217	58	21.1
Time to adequate antibiotics	275	212	63	22.9
**Acute severity markers**				
Peripheral blood WBC	275	192	83	30.2
Initial effluent WBC	275	273	2	0.7
CRP	275	153	122	44.4
Admission / ED / ICU status	275	275	0	0.0

Note: Missingness is summarized for the analytic cohort used in Tables 1–3. For antibiotic timing, missing/not assessable is mainly due to culture-negative episodes, for which organism-level antibiotic adequacy could not be determined.

Abbreviations: APD, automated peritoneal dialysis; CRP, C-reactive protein; ED, emergency department; ICU, intensive care unit; Kt/V, urea clearance index; PD, peritoneal dialysis; RRR, recurrent/relapsing/repeat peritonitis; WBC, white blood cell count.

Supplementary Table S4. Unit-level peritonitis characteristics of the full source cohort by calendar period											
Calendar period	PD patient-years	Clinically treated peritonitis episodes, n	Peritonitis rate, episodes/patient-year	Culture-negative, n (%)	Gram-positive, n (%)	Gram-negative, n (%)	Polymicrobial /fungal /other, n (%)	Effective initial therapy, n (%)	Ineffective initial therapy, n (%)	Not assessable or missing, n (%)	Ineffective among assessable episodes, n/N (%)
2010–2015	878.1	169	0.179	26 (15.4)	62 (36.7)	53 (31.4)	28 (16.6)	101 (59.8)	41 (24.3)	27 (16.0)	41/142 (28.9)
2016–2020	699.5	114	0.156	18 (15.8)	42 (36.8)	33 (28.9)	21 (18.4)	63 (55.3)	34 (29.8)	17 (14.9)	34/97 (35.1)
2021–2025	554.1	102	0.180	24 (23.5)	39 (38.2)	24 (23.5)	15 (14.7)	48 (47.1)	29 (28.4)	25 (24.5)	29/77 (37.7)
Overall	2131.8	385	0.172	68 (17.7)	143 (37.1)	110 (28.6)	64 (16.6)	212 (55.1)	104 (27.0)	69 (17.9)	104/316 (32.9)

Note: Calendar periods were categorized according to the year of the peritonitis episode.

For calculation of peritonitis rates according to the ISPD convention, subsequent relapsing episodes were treated as extensions of the original episode and excluded from the numerator. Twelve, five, and two relapsing episodes were excluded in 2010–2015, 2016–2020, and 2021–2025, respectively, yielding rate numerators of 157, 109, and 100 episodes and an overall numerator of 366 episodes.

Organism groups and initial antibiotic adequacy categories are presented as n (%), with percentages calculated using all clinically treated PD-associated peritonitis episodes within each calendar period as the denominator. Ineffective initial therapy among assessable episodes is presented as n/N (%), where n is the number of episodes with ineffective initial therapy and N is the number of episodes with assessable initial antibiotic adequacy; episodes classified as not assessable or with missing data were excluded.

Abbreviation: ISPD, International Society for Peritoneal Dialysis; PD, peritoneal dialysis.

Supplementary Table S5. Analytic disposition and characteristics of excluded episodes

**Panel A. Analytic disposition and catheter removal according to current RRR status**			
**Characteristic**	**Full source cohort**	**Included analytic cohort**	**Excluded**
Current RRR episodes	65	51	14
Recurrent	6	3	3
Relapsing	19	11	8
Repeat	40	37	3
Catheter removal among current RRR, n/N (%)	13/65 (20.0)	0/51 (0)	13/14 (92.9)
Catheter removal among no current RRR, n/N (%)	94/320 (29.4)	2/224 (0.9)	92/96 (95.8)

**Panel B. Catheter management and disposition of the 110 excluded episodes**			
**Characteristic**	**Overall N=110**	**Current RRR N=14**	**No current RRR N=96**
Peritonitis-associated catheter removal	105 (95.5)	13 (92.9)	92 (95.8)
No peritonitis-associated catheter removal	5 (4.5)	1 (7.1)	4 (4.2)
Peritonitis-associated death	16 (14.5)	2 (14.3)	14 (14.6)

**Panel C. Refractory status and characteristics of excluded refractory episodes**	
**Excluded episodes N=110**	**n (%)**
Refractory status assessable	44 (40.0)
Refractory	42/44 (95.5)
Non-refractory	2/44 (4.5)
Refractory status not assessable	66 (60.0)
**Excluded refractory episodes N=42**	
Gram-positive	10 (23.8)
Gram-negative	22 (52.4)
Culture-negative	4 (9.5)
Polymicrobial/fungal/other	6 (14.3)
Peritonitis-associated catheter removal	41 (97.6)
Peritonitis-associated death	5 (11.9)

Note: Data are presented as n (%) unless otherwise indicated. Current RRR refers to an episode under analysis that was classified as recurrent, relapsing, or repeat in relation to the preceding peritonitis episode. Excluded episodes were those after which PD was not continued and the patient was therefore no longer at risk for a subsequent PD-associated peritonitis episode in the corresponding next-event analysis. In Panel A, catheter removal according to current RRR status is presented as n/N (%), where N is the number of episodes with or without a current RRR phenotype within the corresponding cohort. Two episodes without a current RRR phenotype underwent peritonitis-associated catheter removal but were retained in the analytic cohort because PD was subsequently resumed after catheter reinsertion. In Panel C, refractory and non-refractory percentages were calculated among the 44 excluded episodes with assessable refractory status. Refractory status was considered not assessable when day-5 effluent WBC measurements or sufficient documentation of effluent clearance were unavailable. Microbiology and disposition are presented among the 42 excluded episodes classified as refractory.

Abbreviations: PD, peritoneal dialysis; RRR, recurrent/relapsing/repeat peritonitis; WBC, white blood cell count.

Supplementary Table S6. Follow-up duration, follow-up disposition, and time-based incidence of subsequent peritonitis

Panel A. Follow-up duration and disposition

**Follow-up measure or end status**	**Value**
Eligible episodes	275
Unique patients	152
Follow-up duration, median (IQR), days	294 (66.5–826)
Total episode-time at risk, episode-years	434.5
Subsequent recurrent peritonitis	6 (2.2%)
Subsequent relapsing peritonitis	19 (6.9%)
Subsequent repeat peritonitis	38 (13.8%)
Subsequent non-RRR peritonitis	97 (35.3%)
Death before subsequent peritonitis	32 (11.6%)
Kidney transplantation before subsequent peritonitis	8 (2.9%)
Permanent HD transfer / PD discontinuation before subsequent peritonitis	37 (13.5%)
Transfer to another hospital / loss to follow-up	19 (6.9%)
Administrative censoring	19 (6.9%)

Panel B. Overall and organism-specific incidence rates

Episode under analysis organism group	Episodes	Episode-years at risk	Subsequent RRR events	RRR incidence per 100 episode-years (95% CI)	Subsequent RR events	RR incidence per 100 episode-years (95% CI)
Overall	275	434.5	63	14.5 (11.1–18.6)	57	13.1 (9.9–17.0)
Gram-positive	124	207.9	30	14.4 (9.7–20.6)	26	12.5 (8.2–18.3)
Gram-negative	70	85.9	23	26.8 (17.0–40.2)	21	24.4 (15.1–37.4)
Culture-negative	57	105.8	4	3.8 (1.0–9.7)	4	3.8 (1.0–9.7)
Polymicrobial/fungal/other	24	34.8	6	17.3 (6.3–37.6)	6	17.3 (6.3–37.6)

Note: For each outcome, follow-up ended at the first occurrence of the target subsequent peritonitis event, a competing event, or censoring. Competing events included death, kidney transplantation, permanent transfer to hemodialysis or other permanent discontinuation of PD, and a subsequent peritonitis episode not meeting the target outcome definition. Transfer to another hospital was treated as censoring, and follow-up was administratively censored on December 31, 2025. Incidence rates were calculated per 100 episode-years with exact Poisson 95% confidence intervals.

Abbreviations: CI, confidence interval; HD, hemodialysis; IQR, interquartile range; PD, peritoneal dialysis; RR, relapsing/repeat; RRR, recurrent/relapsing/repeat.

Supplementary Table S7. Separate and mutually adjusted models for APD treatment days and manual exchange burden				
**Variable**	**Separate adjusted OR (95% CI)**	**Separate adjusted P**	**Mutually adjusted OR (95% CI)**	**Mutually adjusted P**
APD treatment days, per 30 days/month	0.42 (0.21–0.83)	0.013	0.93 (0.04–19.29)	0.960
Manual exchange burden, per 30 exchanges/month	1.25 (1.08–1.45)	0.003	1.23 (0.61–2.48)	0.564

Note: APD treatment days and manual exchange burden were strongly inversely correlated (Spearman $\rho=-0.882$; $P<0.001$). Separate models evaluated each prescription variable individually and adjusted for age, sex, diabetes mellitus, and current RRR status. The mutually adjusted model included both APD treatment days and manual exchange burden in addition to the same covariates. All models were fitted using logistic GEE with a binomial distribution, logit link, independence working correlation structure, patient-level clustering, and robust standard errors.

Abbreviations: APD, automated peritoneal dialysis; CI, confidence interval; GEE, generalized estimating equations; OR, odds ratio; RRR, recurrent/relapsing/repeat peritonitis.

Supplementary Table S8. Cross-outcome analyses of patient/PD prescription and acute episode-level predictors

Panel A. Patient-level and PD prescription-related predictors

Variable	Complete-case / assessable N (patients)	Subsequent RRR events	Adjusted OR (95% CI)	Adjusted P	Subsequent RR events	Adjusted OR (95% CI)	Adjusted P
Age, per 10-year increase	275 (152)	63	0.89 (0.73–1.09)	0.247	57	0.86 (0.70–1.06)	0.148
Male sex, vs female	275 (152)	63	0.46 (0.26–0.84)	0.011	57	0.44 (0.23–0.84)	0.013
Diabetes mellitus	275 (152)	63	2.18 (1.14–4.14)	0.018	57	2.12 (1.00–4.49)	0.049
Current episode already RRR	275 (152)	63	3.33 (1.78–6.22)	<0.001	57	3.64 (1.97–6.74)	<0.001
PD vintage, per 1-year increase	274 (152)	63	0.98 (0.90–1.06)	0.607	57	0.98 (0.90–1.06)	0.635
Baseline serum albumin, per 1 g/dL increase	245 (139)	54	0.98 (0.50–1.92)	0.953	48	0.94 (0.46–1.93)	0.876
Peritoneal Kt/V, per 0.5 increase	253 (139)	55	1.52 (0.95–2.42)	0.078	49	1.72 (1.07–2.76)	0.025
Prior exit-site infection	275 (152)	63	1.59 (0.77–3.31)	0.213	57	1.63 (0.79–3.40)	0.188
Prior tunnel infection	275 (152)	63	1.10 (0.54–2.23)	0.800	57	1.21 (0.61–2.41)	0.579
APD treatment days, per 30 days/month	275 (152)	63	0.42 (0.21–0.83)	0.013	57	0.39 (0.19–0.79)	0.009
Manual exchange burden, per 30 exchanges/month	275 (152)	63	1.25 (1.08–1.45)	0.003	57	1.27 (1.09–1.48)	0.002
Glucose load, per 50 g/day	275 (152)	63	0.91 (0.72–1.16)	0.447	57	0.90 (0.72–1.14)	0.387
Any 4.25% dextrose use	275 (152)	63	1.57 (0.64–3.87)	0.327	57	1.88 (0.74–4.77)	0.183
Icodextrin 7.5% use	275 (152)	63	1.39 (0.72–2.67)	0.326	57	1.43 (0.73–2.82)	0.301

Panel B. Acute episode-level predictors

Variable	Complete-case / assessable N (patients)	Subsequent RRR events	Adjusted OR (95% CI)	Adjusted P	Subsequent RR events	Adjusted OR (95% CI)	Adjusted P
Organism group							
Gram-positive	191 (124)	46	Reference	—	41	Reference	—
Gram-negative vs Gram-positive	191 (124)	46	1.77 (0.80–3.90)	0.158	41	1.75 (0.76–4.03)	0.189
Culture-negative vs Gram-positive	191 (124)	46	0.29 (0.09–0.93)	0.038	41	0.39 (0.12–1.25)	0.113
Polymicrobial/fungal/other vs Gram-positive	191 (124)	46	1.26 (0.40–3.99)	0.689	41	1.56 (0.47–5.21)	0.468
Ineffective initial antibiotic therapy vs effective	147 (103)	42	2.71 (1.25–5.86)	0.011	37	3.07 (1.47–6.42)	0.003
Delayed adequate antibiotics ≥1 day after presentation vs same-day	143 (101)	40	2.79 (1.27–6.14)	0.011	35	3.18 (1.44–7.03)	0.004

Peripheral blood WBC, per 1,000/μL increase	191 (124)	46	0.97 (0.89–1.05)	0.427	41	0.94 (0.88–1.01)	0.119
Initial effluent WBC, per doubling	191 (124)	46	0.89 (0.76–1.03)	0.119	41	0.90 (0.76–1.06)	0.200
Admission status, any ED/hospital/ICU vs none	191 (124)	46	1.62 (0.45–5.75)	0.458	41	1.39 (0.39–5.02)	0.613
CRP+1, per doubling	152 (104)	36	1.01 (0.77–1.34)	0.935	31	0.89 (0.67–1.17)	0.405

Note: The broader outcome was subsequent recurrent/relapsing/repeat (RRR) peritonitis, and the narrower outcome was subsequent relapsing/repeat (RR) peritonitis. Each row represents a separate logistic GEE model with a binomial distribution, logit link, independence working correlation structure, patient-level clustering, and robust standard errors. Panel A models included age, sex, diabetes mellitus, and current RRR status, with the predictor itself omitted from the adjustment set when applicable. Panel B models included peripheral blood WBC, $\log_2$-transformed initial effluent WBC, and admission status, with the predictor itself omitted when applicable. Organism-group indicators were entered simultaneously, with Gram-positive episodes as the reference. Initial antibiotic adequacy and delayed adequate therapy were analyzed in separate models. APD treatment days and manual exchange burden were evaluated in separate models because of their strong inverse correlation.

Abbreviations: APD, automated peritoneal dialysis; CI, confidence interval; CRP, C-reactive protein; ED, emergency department; GEE, generalized estimating equations; ICU, intensive care unit; OR, odds ratio; PD, peritoneal dialysis; RR, relapsing/repeat; RRR, recurrent/relapsing/repeat; WBC, white blood cell count.

Supplementary Table S9. Sensitivity analyses of antibiotic-management predictors of subsequent relapsing/repeat peritonitis					
Variable	Complete-case / assessable N	Events	Patients	Adjusted OR (95% CI)	Adjusted P
**Reference model**					
Initial antibiotic ineffective vs effective	147	37	103	3.07 (1.47–6.42)	0.003
Delayed adequate antibiotics ≥1 day after presentation vs same-day	143	35	101	3.18 (1.44–7.03)	0.004
**Restricting to Gram-positive or Gram-negative episodes**					
Initial antibiotic ineffective vs effective	128	32	93	2.89 (1.21–6.92)	0.017
Delayed adequate antibiotics ≥1 day after presentation vs same-day	125	31	92	2.88 (1.14–7.28)	0.025
**Restricting to Gram-positive episodes**					
Initial antibiotic ineffective vs effective	76	16	60	5.92 (1.48–23.63)	0.012
Delayed adequate antibiotics ≥1 day after presentation vs same-day	74	15	59	6.90 (1.52–31.33)	0.012
**Restricting to Gram-negative episodes**					
Initial antibiotic ineffective vs effective	52	16	39	2.29 (0.47–11.21)	0.308
Delayed adequate antibiotics ≥1 day after presentation vs same-day	51	16	39	2.23 (0.39–12.64)	0.365
**Relapsing-only endpoint**					
Initial antibiotic ineffective vs effective	147	13	103	2.42 (0.79–7.41)	0.121
Delayed adequate antibiotics ≥1 day after presentation vs same-day	143	11	101	1.54 (0.40–5.91)	0.529
**Repeat-only endpoint**					
Initial antibiotic ineffective vs effective	147	24	103	2.64 (1.14–6.11)	0.023
Delayed adequate antibiotics ≥1 day after presentation vs same-day	143	24	101	3.56 (1.51–8.35)	0.004

Note: The narrower outcome was subsequent relapsing/repeat peritonitis. Each row represents a separate logistic GEE model with binomial distribution, logit link, independence working correlation structure, patient-level clustering, and robust standard errors, unless otherwise specified. The core adjusted model included peripheral blood WBC count, $\log_2$-transformed initial effluent WBC count, and admission status. Initial antibiotic adequacy and time to adequate antibiotics were analyzed in separate models. Organism-specific subgroup analyses were exploratory and limited by small event counts. The wide confidence intervals indicate substantial imprecision; therefore, the magnitude of the subgroup-specific estimates and differences between organism groups should be interpreted cautiously.

Abbreviations: CI, confidence interval; GEE, generalized estimating equations; OR, odds ratio.

**Supplementary Table S10. Sensitivity analyses omitting peripheral blood WBC from models of subsequent relapsing/repeat peritonitis**			
**Predictor**	**Sensitivity N**	**Adjusted OR (95% CI)**	**P**
Ineffective initial therapy vs effective	216	3.00 (1.57–5.72)	<0.001
Delayed adequate therapy vs same-day	211	3.27 (1.65–6.49)	<0.001
Initial effluent WBC, per doubling	273	0.94 (0.80–1.11)	0.466
Admission status, any ED/hospital/ICU vs none	273	1.03 (0.55–1.94)	0.916
CRP+1, per doubling	152	0.88 (0.67–1.16)	0.371

Note: Each row represents a separate logistic GEE model for subsequent relapsing/repeat peritonitis. Sensitivity models omitted peripheral blood WBC from the adjustment set and included log_2-transformed initial effluent WBC and admission status, except when the covariate itself was the predictor of interest. Initial antibiotic adequacy and delayed adequate therapy were evaluated in separate models.

Abbreviations: CI, confidence interval; CRP, C-reactive protein; ED, emergency department; GEE, generalized estimating equations; ICU, intensive care unit; OR, odds ratio; WBC, white blood cell count.

Supplementary Table S11. Calendar-period adjusted sensitivity analyses					
**Outcomes and predictor**	**Complete-case / assessable N**	**Events**	**Patients**	**Calendar-period adjusted OR (95% CI)**	**Calendar-adjusted P**
Subsequent RRR peritonitis					
Male sex	275	63	152	0.46 (0.26–0.82)	0.009
Diabetes mellitus	275	63	152	2.70 (1.33–5.50)	0.006
Current RRR phenotype	275	63	152	3.25 (1.70–6.20)	<0.001
Peritoneal Kt/V, per 0.5 increase	253	55	139	1.81 (1.08–3.03)	0.023
APD treatment days, per 30 days/month	275	63	152	0.52 (0.26–1.05)	0.067
Manual exchange burden, per 30 exchanges/month	275	63	152	1.18 (1.02–1.38)	0.031
Subsequent RR peritonitis					
Ineffective initial antibiotic therapy vs effective	147	37	103	3.02 (1.44–6.34)	0.003
Delayed adequate antibiotics ≥1 day after presentation vs same-day	143	35	101	3.28 (1.48–7.26)	0.003

Note: Calendar period was categorized according to the year of the episode under analysis as 2010–2015, 2016–2020, and 2021–2025. For the broader RRR outcome, calendar period was added to the core adjusted models that included age, sex, diabetes mellitus, and current RRR phenotype, with the predictor itself omitted from the adjustment set when applicable. For the narrower RR outcome, calendar period was added to the core adjusted models that included peripheral blood WBC count, log_2-transformed initial effluent WBC count, and admission status. Each row represents a separate logistic GEE model with patient-level clustering and robust standard errors.

Abbreviations: APD, automated peritoneal dialysis; CI, confidence interval; GEE, generalized estimating equations; OR, odds ratio; RR, relapsing/repeat; RRR, recurrent/relapsing/repeat; WBC, white blood cell count.
